# 

**Published:** 1913-07

**Authors:** 


					﻿®	od64iv4-state soc/efy soc/a/ wyg/ene and r/^en/cs. |
Vol. XXIX.
JULY, 1913
IO#I
PgRiHBa^’B
jeaas medical jouk al-
\	%	ESTABLISHED JULY, 1885 / ' '	-	\	.
\	PUBLISHED MONTHLY AT AUSTIN, TEXAS, BY
- , -EE.DANIEL, M. D., - - - ’ Editor and Proprietor.;
PRINTED BY THE VON BOECKAfANN-JONES CO.
a
■
. ft
i’w
IE
z
z
Hl’ .■
IM
h
Z
Subscription, $1.00 a Tear, in Advance. -	-	-	- Single Copies. 10 Cents
Entered at the Postoffice at Austin, Texas, as second class mail mattaa.—
				

